# The Modern Treatment of Syphilitic Diseases, Both Primary and Secondary

**Published:** 1846-04

**Authors:** 


I. The Modern Treatment of Syphilitic Diseases, both Primary and Secondary: comprising numerous Formulæ for the Preparation and Mode of Administration of the New Remedies; and an Account of a safe and successful Mode of Treating Chronic, Protracted, and Constitutional Syphilis, by the Mercurial Vapour Bath. By *Langston Parker*, Surgeon to the Queen's Hospital, Birmingham, &c. Second Edition, 12mo. pp. 228. London, Churchill, 1845.

II. Lettre sur la Syphilis. Par *S. Ratier*. 8vo. pp. 68. Paris, 1845.

A Letter on Syphilis. By *S. Ratier*.

We rather fully reviewed the former edition of Mr. Parker's work in our January number of 1840. The author remarks, in his Preface, that six years' additional experience, both in hospital and private practice, has enabled him to confirm the efficacy of most of the plans of treatment recommended in the first edition of this work. Much new matter has been added in the present edition. *That which he regards of the first importance is the account of the treatment of various forms of syphilis by the mercurial vapour-bath.* Diseases rebellious or tedious under ordinary treatments, generally yield with ease to this combination; more particularly affections of the skin and bones; the duration of treatment by it is much shortened, the quantity of medicine required to be given by the mouth greatly diminished, and the cures rendered more permanent and certain. Relapses after this mode of treatment Mr. Parker has found extremely rare; whereas, under the ordinary plan, they are exceedingly frequent, even after a perfect cure had been supposed to have been effected. In the Chapter on Mercurial Fumigations, he states, the method employed by Werneck, in Germany, appears to him the simplest and most efficacious, and is that which he has recourse to. The mode is thus described :—

"The patient is prepared for the fumigations by low diet, the warm bath used daily, aperients, and the compound decoction of sarsaparilla. This preparatory treatment is pursued for five or six days prior to the fumigation, and the patient is confined to his room, which should be kept moderately warm. At the end of this period a sharp aperient should be exhibited, and the fumigation may be then employed. The patient is placed upon a seat, covered with a mantle of waxed or oiled cloth; the apparatus, which consists merely of a spirit lamp and a china plate, upon which the cinnabar or bisulphuret of mercury is laid, put under it. The mantle should be fixed closely round the neck, to prevent the mercurial vapours escaping into the room.

"The fumigation is continued for a quarter of an hour, in a warm chamber, and, at its conclusion, the patient is directed to go to bed; it is for this reason that the remedy is most conveniently employed in the evening. The quantity of cinnabar necessary for each fumigation is from twenty grains to a drachm; one application a day is sufficient, and the cure is generally complete in eighteen or twenty days; in case of salivation occurring, or any circumstances affecting the constitution of the patient, the fumigations must be used less frequently, the quantity of the mercury diminished, or the remedy altogether discontinued. When the treatment is terminated, the patient is to change his linen and take a bath." P. 197.

The reader, after perusing the above description, will be at a loss to perceive any novelty justifying the promise in the Preface which we have given in Italics. We had really supposed that Mr. Parker had quite forgotten the new matter which was of the first importance, until, on arriving at the last page but one in his work, we found the following passage:

"I have adopted, however, a method somewhat different to any I have hitherto seen used; it consists in the application of mercurial vapour in a moist state to the surface of the skin, combining, in fact, the mercurial fume with the ordinary vapour-bath. I have made a number of experiments on this combination, and found it succeed in a variety of cases, where ordinary mercurial fumigation, or the vapour-bath, employed separately, had failed." P. 220.

The author states that, he could bring forward many cases as evidence of the efficacy of the baths. In one, the patient had undergone a regular mercurial treatment for a primary sore, followed by an excavated ulcer of the tonsil, and a scaly eruption; he was profusely salivated, and kept the house for a month. Two months after he supposed himself cured, he became again covered with a similar eruption, which entirely and permanently disappeared under the use of baths and very minute doses of the bichloride of mercury with guaiacum. No account is given of the period during which the baths were used.

In a second case, a gentleman of large fortune,* had an indurated cica-

* What this gentleman's "large fortune" had to do with his indurated chancre trix of six months' standing (a symptom confessedly difficult to remove) with an excavated ulcer of one tonsil, of several weeks' standing; he had been taking, under an eminent surgeon, five grains of calomel, and as much blue pill, night and morning, for three weeks, without the slightest influence upon the disease, which entirely disappeared under three weeks' treatment by the baths, and nightly inunctions of a very small quantity of mercurial ointment. This patient was not confined during the treatment. That a syphilitic induration of six months' standing, entirely disappeared under three weeks' treatment by the baths and nightly inunctions, we wholly disbelieve; at any rate, we should attribute a good deal to the influence which had already been produced on the system by the previous three weeks' course of calomel and blue pill. The particulars of these cases are meagre enough, and they are far from convincing us of the great benefit of this mode of treatment. Certainly those who, after reading the Preface to this edition of the work, may naturally expect to find a satisfactory account of the treatment of syphilis by the mercurial vapour-bath, will be disappointed, and if any should purchase the book on the faith of this promise, they will not be pleased to read the announcement on another page, that the author is preparing for publication another work illustrative of the efficacy of this plan of treatment.

The objection to mercurial fumigations made long ago by Mr. Pearson, that it was difficult by this means to regulate the action of the mercury, is the opinion entertained by the best practical surgeons of the present day; and we believe that the objection equally applies to the mercurial vapour-bath, and it must prevent this remedy becoming so generally applicable as Mr. Parker's favourable report would seem to indicate.

We cannot perceive that this work contains much new matter. It is, as we remarked in our previous review, mainly a compilation from French writers, with the addition of cases occurring in the author's own practice.

II. M. Ratier, from the year 1828, has paid great attention to the subject of Syphilis, having been an assiduous observer in the wards of the late M. Cullérier, and a *collaborateur* of that eminent practitioner, in the articles contributed by him on the subject to the *Dictionnaire de Medicine et de Chirurgie Pratiques.* He has diligently perused the works of all his predecessors, and now puts forth his own views, in the modest *brochure* before us. We feel pleasure in laying the conclusions the author has arrived at before our readers, not that we can by any means agree to their general correctness, but from our willingness to aid the more extensive enquiries upon the subject, which he contemplates making, and our entire sympathy with the censures he passes upon the charlatanism and polypharmacy with which practitioners are too often wont to invest the management of this disease.

According to M. Ratier, *chancre* is the sole primary symptom of syphilis, which, left to Nature, terminates with hygienic cares, within 40 days. It may be accompanied by various accidental phenomena, such as bubo,

---

we cannot tell. When will authors cease to gratify their vanity by parading the titles and wealth of their patients. To say the least, it exhibits very bad taste.

vegetations, &c., which, however, are no essential features of the disease. This chancre may be cured without leaving any traces of its existence, or giving rise to any general infection of the system, although it might have been propagated had it been inoculated. *Constitutional syphilis* usually follows the chancre, although it sometimes appears simultaneously with it. It is improperly so named; for it is, properly speaking, only an affection of the *skin* and *mucous membranes*, to which M. R. gives the appellation of *papular syphilide* or *syphilitic papula*. This is so characteristic, that, if a few inches of the skin can be seen, the nature of the case may be pronounced upon. Its duration is indeterminate, it is susceptible of perfect cure, and is not contagious like primary syphilis. When neglected, it may lead to ulcerations of various parts wherein it is located, and thus give rise to various appearances which are unnecessarily subdivided into varieties of syphilis by authors. So little difficulty does he attach to the management of this disease, that the author declares he would much prefer being the subject of it than of the fracture of a bone.

In treating the disease, M. Ratier does not employ *mercury* for the *primary* sore, and therefore not necessarily at all. He says—

"If I happened to contract syphilis, I should commence by cauterizing the pustule, so as to convert what might prove a virulent ulcer into a simple wound, which would cicatrize in the ordinary space of time a simple sore requires. If I had not commenced the treatment sufficiently early, or the cauterization had not succeeded, I should abandon the ulcer to itself, taking care to abstract from it whatever could add an accessory inflammation to that which is essential to the disease, as also abstaining from all emollient and relaxing applications, which are as injurious in another way. I should confine myself to a moderate regimen, such as reasonable healthy persons adopt, and continue my ordinary occupations, awaiting the event in entire security. The only precaution I should take, would be to proportion the number of dressings to the amount of discharge, and to very carefully cleanse each of the ulcers and neighbouring surfaces, and to cover them with soft and dry lint, which easily absorbs the secreted fluids. By such means, many experiments have shewn me that the ulcers would become cicatrized by the end of *forty days*. I admit that, through neglect of the dressings, errors of regimen, or fatiguing exertions, swelling of the glands in the vicinity of the ulcers might occur. In this case I should rest, adopt an abstinent regimen, and, by redoubled care in dressing the primary sores, I should arrest absorption, and in a few days the swelling would dissipate. But suppose it to have been neglected and suppuration has occurred, I should let out the matter as soon as formed, applying leeches and emollients in the vicinity, and be especially careful in dressing the chancre, which last is a *sine quâ non*.

"During this time, or sooner or later after the chancre is cicatrized, but usually not longer after, a *papular eruption* may shew itself on various parts of the body. This phenomenon advertizes me of my true position, and, without being rendered uneasy by it, I at once commence the specific treatment by mercury, which is the most certain means of obtaining a prompt and durable cure. * * * * It is to be well understood, then, that I only have recourse to the mercurial treatment in constitutional syphilis, and never for a primary sore.

"What I have here stated hypothetically is my habitual practice. When the patient is cured completely of his chancre, I consider him free from ulterior accidents. If there are glandular swellings, I entertain a suspicion of his condition for five or six months, this being the longest period during which symptoms of general infection usually exhibit themselves. Lastly, after the complete disappearance of the papular syphilide, whether this has taken place spontaneously, or resulted from the use of mercury, I consider the patient quite cured, and exempt from all future accidents, unless there has been a new inoculation."

The author severely criticizes the confused ideas which have prevailed in reference to the management of this disease, especially by mercury. "In the *Pharmacopée Universelle* of M. Jourdain there are at least 800 modes of administering mercury mentioned, each of which is better than any that has preceded it." Some eminent practitioners entirely neglect local treatment. Some think a very mild course of mercury is only required, others a complete one, and others, again, that the disease can never be eradicated. Silver, gold, iodine, tartar-emetic, and a variety of other substances, have been proposed as substitutes for mercury—so that, amid so many novelties, we may sometimes exclaim in the words of Boyer, "Let us make and use this remedy while it will still cure the affection." As, already observed, he much objects to dressing the chancre with mercurial ointments, &c., which, in fact, only increase the loss of substance; as also with emollients which, by diluting the morbid secretions, diffuse them over a larger space, and render them more susceptible of absorption. Stimulant applications may prove beneficial by changing the mode of action of parts, and by diminishing absorption by the irritation they produce. This explains the success which often follows treating chancre by superficial applications of *nitrate of silver*, renewing these as often as the eschar falls off. The practice of *inoculation* he considers as highly reprehensible, in multiplying the chances of general infection, and as nowise necessary for determining the treatment to be followed. Of all the preparations of *mercury* in the cases where it is required, he prefers the *deuto-chloride*. Although after a few days the papules will have ceased to appear and have become diminished in size, two months will be required to entirely get rid of them. Administered by careful hands, mercury is entirely exempt from the evils so commonly attributed to it. It is hurtful, however, when continued for long after the symptoms requiring it have disappeared. The abuse of mercurials heretofore gave reputation to the *sudorifics*, (which however do not cause sweating here,) which prove useful rather from the suspension of the mercury, and the attention to regimen, than from any efficacy of their own. As to *gold* and *iodine*, they have not cured primary chancre, but have only allowed it to cure itself; and in constitutional syphilis they have either succeeded after the excessive employment of mercury, or have been used in *pseudo-syphilitic* cases, mistaken for syphilis. For the local treatment of the eruption in constitutional syphilis, and the ulcerations, &c., it may give rise to—

"The best means I have found to be superficial *cauterization with the nitrate of silver*. It covers the parts with an immoveable protecting layer, and is far easier of application than any form of dressing. It must be renewed wherever the eschar falls off. No one would believe the beneficial effects it produces on the red, inflamed, and suppurating parts, to which, according to the usual plan, we should apply emollients. But, however useful an adjuvatory means this may prove, it does not suffice for cure, it does not prevent the occurrence of new papules; and, in order to lose no time, we should at once have recourse to mercurial treatment, which, if it be not the only means of cure, is at least the one that effects its object soonest and most certainly. I need enter into no details as to the employment of the cauterization, but may just repeat what I have elsewhere stated of its great utility in syphilitic *onychia*—a simple, painless, treatment, capable of substitution for the barbarous practice of tearing off the nail.

"Vegetations, excrescences, bubos, and the indurated ulcers which succeed to them, the various cutaneous affections, exostoses, and the periosteal pains, which coincide with primary or constitutional syphilis, are not of the same nature, although often cured during its treatment. More frequently they persist, in spite of the disappearance of the characteristic symptoms of the disease, and then they should be attacked separately and locally. To renew the mercurial treatment, over and over again, as was the fashion sometime since, is a foolish and dangerous practice."

In his dislike of the present complicated and uncertain views respecting the nature and treatment of Syphilis, M. Ratier seems to us to invest these with a greater degree of simplicity than our present knowledge justifies our attaching to them. The indefinite multiplication of species and varieties is bad, but surely we are not in a condition to admit that the whole evidence of the disease is comprised in a primary sore and a papular eruption. That there may be general infection without any such eruption manifesting itself, we can have no doubt; and the recommendation to withhold mercury until such appears, seems to us a very dangerous one, incurring as it does the loss of much valuable time, and the running needless risk. That sores *can* be healed without mercury has been proved times out of number, but the withholding it at the commencement of the disease, to administer it only upon this cutaneous affection appearing, is very questionable practice. Then, again, we are by no means disposed to agree with the author in the low estimate he forms of Iodine and its various invaluable preparations. However, M. Ratier is a man of large experience, writes in a candid spirit, and seeks for criticism friendly or adverse, expressing his willingness to explain more fully whatever may appear obscure in, or inconsistent with his doctrine.

